# Stimulation of Perforant Path Fibers Induces LTP Concurrently in Amygdala and Hippocampus in Awake Freely Behaving Rats

**DOI:** 10.1155/2013/565167

**Published:** 2013-01-17

**Authors:** J. Harry Blaise, Rachel A. Hartman

**Affiliations:** ^1^Neuroscience Program, Trinity College, 300 Summit Street, Hartford, CT 06106, USA; ^2^Department of Engineering, Trinity College, 300 Summit Street, Hartford, CT 06106, USA

## Abstract

Long-term potentiation (LTP) which has long been considered a cellular model for learning and memory is defined as a lasting enhancement in synaptic transmission efficacy. This cellular mechanism has been demonstrated reliably in the hippocampus and the amygdala—two limbic structures implicated in learning and memory. Earlier studies reported on the ability of cortical stimulation of the entorhinal cortex to induce LTP simultaneously in the two sites. However, to retain a stable baseline of comparison with the majority of the LTP literature, it is important to investigate the ability of fiber stimulation such as perforant path activation to induce LTP concurrently in both structures. Therefore, in this paper we report on concurrent LTP in the basolateral amygdala (BLA) and the dentate gyrus (DG) subfield of the hippocampus induced by theta burst stimulation of perforant path fibers in freely behaving Sprague-Dawley rats. Our results indicate that while perforant path-evoked potentials in both sites exhibit similar triphasic waveforms, the latency and amplitude of BLA responses were significantly shorter and smaller than those of DG. In addition, we observed no significant differences in either the peak level or the duration of LTP between DG and BLA.

## 1. Introduction

Long-term potentiation (LTP), a form of synaptic plasticity, is an activity-dependent increase in synaptic strength induced by high frequency stimulation of afferent pathways [1]. Owing to its associativity, specificity, and persistence properties LTP is now widely considered as a cellular model for learning and memory [2–5]. Much attention in LTP research has focused on the hippocampus which is thought to be involved in learning and memory processes. More recently, the amygdala has enjoyed renewed interest due to its implication in modulating synaptic plasticity in the hippocampus, the prefrontal cortex, and the anterior cingulate cortex [6–9] and its involvement in memory consolidation and emotional memories [10–17]. 

Emotional memories, including fear conditioning and extinction, are thought to be mediated by the amygdala [10, 18–21]. The basolateral amygdala (BLA) in particular has been shown to be extensively connected with cortical and subcortical structures involved in memory and emotion, notably the hippocampal formation, the striatum, the prefrontal cortex, the entorhinal cortex, the association cortices, and the thalamus, among others [22, 23]. As a result, the BLA is thought to play a crucial role in psychophysiological responses to emotionally salient events or sensory stimuli that trigger emotional arousal [22, 24, 25]. 

While there have been many reports indicating reciprocal connections between entorhinal cortex (EC) and both DG and BLA very few studies have investigated simultaneous LTP induction in both hippocampus and amygdala. Among these are studies that have utilized cortical stimulation of the EC [26–28] or animals under acute anesthesia [26, 28]. On the one hand, differences in neuronal responses exist between cortical and fiber stimulation of afferents [29]. On the other hand, acute anesthesia has been shown to alter central inhibition and/or tonic excitability of the neuronal population under study [30–32]. Moreover, the majority of in vivo studies of LTP in the DG have focused on activation of perforant path fibers, not cortical stimulation of the EC [33–36]. Therefore, to retain a stable baseline of comparison with the majority of the LTP literature and to circumvent the intrinsic limitations of in vitro and acute anesthetized preparations, the present study examines concurrent induction of LTP in the DG and the BLA following theta burst stimulation of perforant path fibers in freely behaving rats.

## 2. Materials and Methods

All surgical and experimental protocols were approved by the Trinity College Animal Care and Use Committee and were in accordance with the NIH Guide for the Care and Use of Laboratory Animals. Details of our electrophysiological procedures in freely behaving rats were published previously [37]. But briefly, under IP-administered sodium pentobarbital anesthesia (50 mg/kg), male Sprague-Dawley rats (70–90 days of age) were chronically implanted with a concentric bipolar stimulating electrode positioned in the angular bundle (AP: −7.6 mm; LAT: +4.3 mm; DV: −2.5 mm relative to Bregma) to activate perforant path fibers; single-strand tungsten wire recording electrodes positioned in the DG (AP: −4.0 mm; LAT: +2.5 mm; DV: −3.1 mm) and the BLA (AP: −3.1 mm; LAT: +5.0 mm; DV: −7.8 mm). Final dorsoventral positioning of electrodes was achieved by visual observation of the maximum negative-going population spike amplitude at the lowest stimulus intensity for both DG and BLA. The electrode wires were then led to a contact pin headstage assembly which was fixed to the skull with fast-drying dental acrylic (Lang Dental Manufacturing, Wheeling, IL).

Following a one-week postsurgery recovery period, animals were placed in a shielded recording chamber and connected to the recording equipment via low-noise shielded cables attached to a commutator assembly (Plastics One, Roanoke, VA). This system allowed free and unimpeded movement of behaving animals in the recording chamber. All animals were allowed 5 hours acclimation and habituation time in the chamber prior to commencement of experiments. Electrical stimulation was provided by a Grass S-88 stimulator and consisted of biphasic square-wave pulses (pulse width = 0.25 ms, 50% duty-cycle) passed through a pair of Grass PSIU-6 photo stimulus isolation units to provide constant current (Astro-Med Inc., West Warwick, RI). Evoked responses were amplified (gain = 1000), and bandpass filtered from 1 Hz–3 KHz (DAM-50, World Precision Instr., Sarasota, FL), passed to a digital oscilloscope (Integra 15, Nicolet Instruments, Madison, WI) for visual inspection, digitized (LabVIEW, sampling rate = 70 KHz, 12-bit resolution), and stored on computer for field potential analysis.

## 3. Results

**Figure 1:**
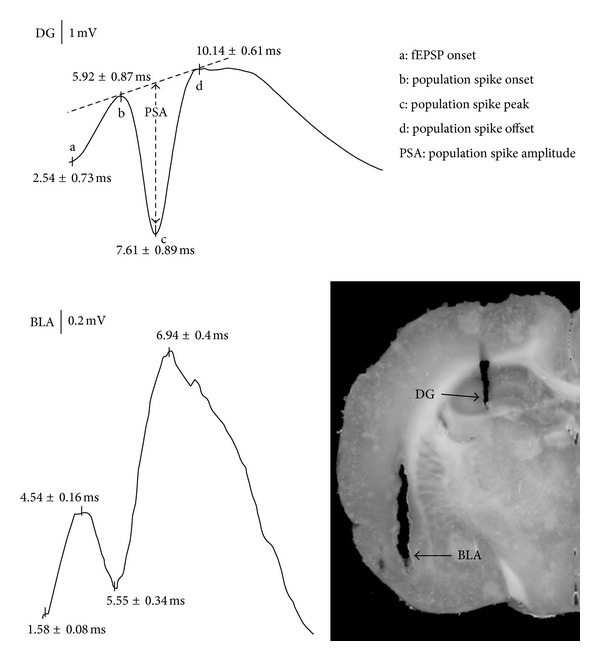
Representative traces of evoked field potentials recorded in BLA and DG in response to stimulation of perforant path fibers. Also shown are methods for quantifying the amplitude of the population spike (PSA), as well as histological confirmation of electrode placement in BLA and DG.

Each animal was stimulated with a current intensity equal to 50% maximal response as determined by that animal's DG input/output (I/O) curve (typical current ranges from 400 to 1400 uA). With these parameters, the excitatory postsynaptic potential in DG is usually contaminated by the population spike; thus we limited our analysis to population spike amplitude with respect to both sites. Since it has been shown that hippocampal evoked potentials vary with vigilance state, all experiments were conducted during times when animals were in the state of quiet waking which is characterized behaviorally by animals lying on the floor of the cage posturally relaxed with eyes open and electrographically by desynchronized EEG activity with occasional low amplitude spindles and delta waves [38, 39]. Single-pulse stimulation delivered to perforant path fibers resulted in simultaneous recordings of triphasic field evoked potentials in both the BLA and the DG. Representative traces of these evoked field potentials along with the methods used to quantify the population spike amplitude (PSA) appears in Figure 1. All amplitude values were subjected to statistical analysis using a repeated-measures ANOVA. Significant main effects (*P* < 0.05) were further analyzed post hoc using a student Newman-Keuls test. 

**Figure 2:**
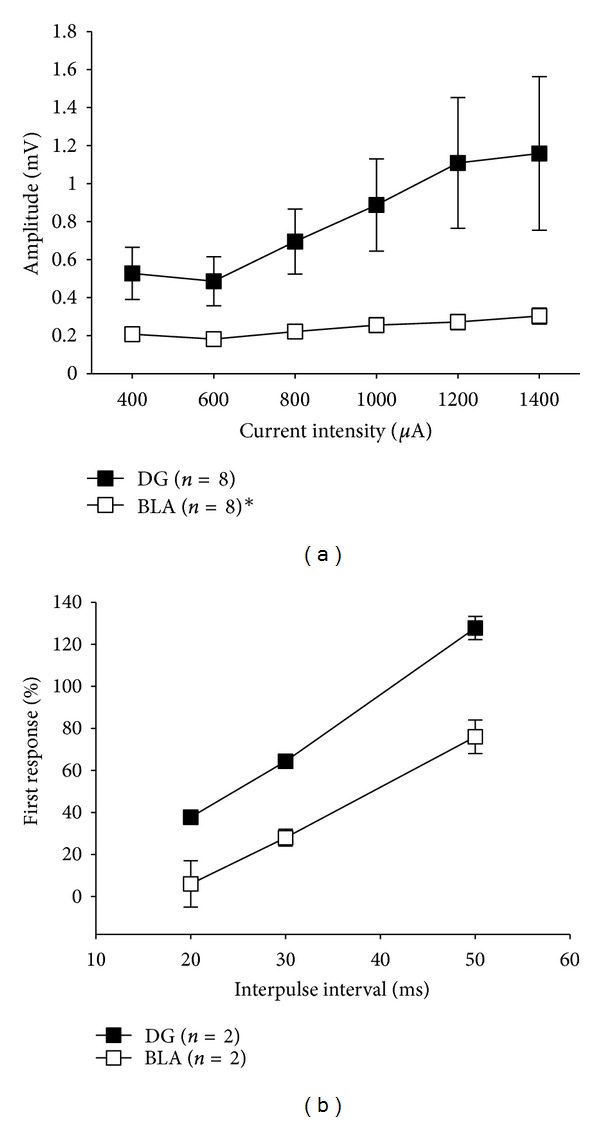
(a) Mean (±SEM) baseline input/output curves recorded in DG and BLA. DG neurons are significantly more excitable than BLA (*P < 0.05). (b) Responses to paired-pulse stimuli show markedly greater paired-pulse inhibition in BLA for short latencies (20 and 30 ms).

Although evoked potentials at both sites appeared to have similar triphasic waveforms consisting of a positive fEPSP followed by a negative population spike, shorter peak latency, and smaller spike amplitude were observed in BLA compared to DG (Figure 1). For example, the fEPSP onset was measured at 1.58 ms in the BLA compared to 2.54 ms in the DG. Similarly, the latency of the negative-going peak in the BLA response was measured at 5.55 ms compared to 7.61 ms in the DG. These results differ from previous studies reporting much longer peak latency and a monophasic waveform morphology for both BLA and DG responses [27, 28]. This difference may be attributed to the cortical stimulation paradigm used in the previous studies compared to perforant path fiber stimulation used in the present study. Remarkably, our results show that prior to tetanization the I/O curve recorded in the BLA was significantly (*P* < 0.05) lower in amplitude and remained relatively flat across increasing stimulation current compared to that recorded in the DG (Figure 2(a)). Specifically, evoked responses to low intensity stimulation (400 uA) averaged 0.2 mV in the BLA compared to 0.5 mV in the DG and 0.3 mV in BLA versus 1.2 mV in DG for higher intensity stimulation (1400 uA) (Figure 2(a)). In another series of experiments, we recorded paired-pulse responses to short latency paired-stimulus intervals (20, 30, and 50 ms) to ascertain the nature of BLA inhibition. As shown in Figure 2(b), 20 and 30 ms paired-stimulus intervals resulted in greater inhibition of the response to the second pulse in BLA compared to DG.

**Figure 3:**
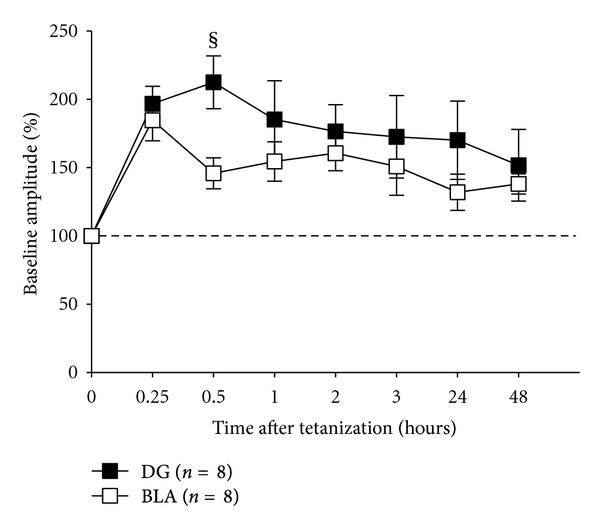
Theta burst stimulation of the perforant path results in LTP recorded concurrently in BLA and DG. Analysis of variance revealed no significant difference between DG-LTP and BLA-LTP at any of the time points (^§^ *P* > 0.11). Each point represents the average of 5 responses to single-pulse stimulation recorded in each animal (stimulation frequency = 0.1 Hz).

Regarding concurrent LTP in DG and BLA, 5-Hz theta burst stimulation (10 bursts of 10 pulses delivered at 400 Hz with a burst rate of 5 Hz) of perforant path fibers resulted in recording of potentiated field potentials in both sites (Figure 3). In effect, we observed enhanced LTP of similar elevation in both sites with peak potentiation of +196.6 ± 14.8% in the DG and +184.6 ± 16.2% in the BLA. Statistical analysis revealed no significant differences in either the peak or the duration of LTP between DG and BLA (*P* > 0.11). Nevertheless, LTP persisted for at least 48 hours in both structures (DG: +151.8 ± 28.1%; BL: +138.0 ± 7.9%) (Figure 3). 

## 4. Discussion

This study which was designed to investigate perforant path fiber-induced concurrent LTP in the DG and the BLA discovered three important results. First, as shown in Figure 1, although evoked potentials at both sites exhibit a similar triphasic waveform, the latencies and amplitudes are significantly shorter and smaller in BLA relative to DG. This finding is consistent with results of previous studies showing a marked reduction of field potential amplitude in BLA in response to external capsule stimulation [40]. Second, as indicated in Figure 2(a), the BLA I/O curve is of significantly lower amplitude and stayed flat across increasing input current intensity. In another series of experiments, paired-pulse responses recorded in BLA and DG show a marked difference in inhibition of the second pulse (Figure 2(b)). Paired-pulse inhibition was greater in BLA than DG. These findings suggest that BLA tonic neuronal excitability may have a narrower dynamic range compared to DG. This may be due to extensive inhibitory control exerted on the BLA by noradrenergic and glutamatergic modulation of GABA release mediated by *α*_1A_ adrenoreceptors and GluR5 kainate receptors, respectively [41–46]. This strict inhibitory regulation of the BLA may subserve its ability to adequately process and respond to stressful and emotionally significant stimuli by reducing the likelihood of BLA overexcitation during periods of stress. This mechanism would appear to underlie coping strategies used to counteract some of the pathophysiological changes observed in mood and anxiety disorders such as major depressive disorders, posttraumatic stress disorders, and perhaps even status epilepticus [45]. 

Lastly, our results of enhanced LTP of similar level in both sites (Figure 3) are in agreement with previous studies of simultaneous LTP in the DG and BLA following cortical theta burst stimulation of the EC [27, 28]. Nonetheless, our results also differ from the above studies in that we observed smaller evoked response amplitude in the BLA than that reported by either Yaniv and colleagues and Vouimba et al. This divergence may be attributed to the different electrophysiological approaches used in the present study compared to the previous studies: fiber versus cortical stimulation; freely behaving versus acute anesthetized animals; and triphasic versus monophasic waveforms of evoked responses. Thus, our results seem to indicate that while tonic neuronal excitability is regulated differentially in BLA and DG, there is no difference in long-term synaptic plasticity.

In conclusion, in the present study we have tested the reliable induction and maintenance of concurrent LTP in the BLA and the DG following fiber activation of the perforant path in freely behaving rats, and we have compared these results with cortically induced LTP. Nevertheless, we cannot completely rule out that stimulation of the perforant path at the angular bundle may have concurrently activated separate afferents from the entorhinal cortex to the amygdala, particularly afferents from layers V and VI of the lateral entorhinal cortex [47]. Future studies are needed to determine whether the PP-BLA connection is monosynaptic, polysynaptic, or a result of entorhinal cortex projections to the BLA through the ventral angular bundle [48]. Even so, it appears that the BLA is under strict inhibitory control—a mechanism which may promote coping strategies in anxiety disorders, major depressive disorders, and status epilepticus caused by putative BLA overexcitation.

## References

[1] Bliss TVP, Lomo T (1973). Long lasting potentiation of synaptic transmission in the dentate area of the anaesthetized rabbit following stimulation of the perforant path. *Journal of Physiology*.

[2] Martin SJ, Grimwood PD, Morris RGM (2000). Synaptic plasticity and memory: an evaluation of the hypothesis. *Annual Review of Neuroscience*.

[3] Bliss TVP, Collingridge GL (1993). A synaptic model of memory: Long-term potentiation in the hippocampus. *Nature*.

[4] Malenka RC, Nicoll RA (1999). Long-term potentiation—a decade of progress?. *Science*.

[5] Abraham WC, Williams JM (2003). Properties and mechanisms of LTP maintenance. *Neuroscientist*.

[6] Ikegaya Y, Saito H, Abe K (1995). High-frequency stimulation of the basolateral amygdala facilitates the induction of long-term potentiation in the dentate gyrus in vivo. *Neuroscience Research*.

[7] Akirav I, Richter-Levin G (2002). Mechanisms of amygdala modulation of hippocampal plasticity. *Journal of Neuroscience*.

[8] Bush G, Luu P, Posner MI (2000). Cognitive and emotional influences in anterior cingulate cortex. *Trends in Cognitive Sciences*.

[9] Davis M, Hitchcock JM, Bowers MB, Berridge CW, Melia KR, Roth RH (1994). Stress-induced activation of prefrontal cortex dopamine turnover: blockade by lesions of the amygdala. *Brain Research*.

[10] LeDoux JE (2000). Emotion circuits in the brain. *Annual Review of Neuroscience*.

[11] McGaugh JL (2002). Memory consolidation and the amygdala: a systems perspective. *Trends in Neurosciences*.

[12] McGaugh JL (2004). The amygdala modulates the consolidation of memories of emotionally arousing experiences. *Annual Review of Neuroscience*.

[13] Paré D (2003). Role of the basolateral amygdala in memory consolidation. *Progress in Neurobiology*.

[14] Roozendaal B (2003). Systems mediating acute glucocorticoid effects on memory consolidation and retrieval. *Progress in Neuro-Psychopharmacology and Biological Psychiatry*.

[15] Cahill L, McGaugh JL (1996). Modulation of memory storage. *Current Opinion in Neurobiology*.

[16] McIntyre CK, Miyashita T, Setlow B (2005). Memory-influencing intra-basolateral amygdala drug infusions modulate expression of Arc protein in the hippocampus. *Proceedings of the National Academy of Sciences of the United States of America*.

[17] Eichenbaum H, Otto T, Cohen NJ (1992). The hippocampus—what does it do?. *Behavioral and Neural Biology*.

[18] Maren S (2001). Nuerobiology of Pavlovian fear conditioning. *Annual Review of Neuroscience*.

[19] McGaugh JL, McIntyre CK, Power AE (2002). Amygdala modulation of memory consolidation: interaction with other brain systems. *Neurobiology of Learning and Memory*.

[20] LaBar KS, Cabeza R (2006). Cognitive neuroscience of emotional memory. *Nature Reviews Neuroscience*.

[21] Phelps EA, Delgado MR, Nearing KI, Ledoux JE (2004). Extinction learning in humans: role of the amygdala and vmPFC. *Neuron*.

[22] Mcdonald AJ (1998). Cortical pathways to the mammalian amygdala. *Progress in Neurobiology*.

[23] Canto CB, Wouterlood FG, Witter MP (2008). What does the anatomical organization of the entorhinal cortex tell us?. *Neural Plasticity*.

[24] Packard MG, Cahill L (2001). Affective modulation of multiple memory systems. *Current Opinion in Neurobiology*.

[25] Cahill L, McGaugh JL (1998). Mechanisms of emotional arousal and lasting declarative memory. *Trends in Neurosciences*.

[26] Kavushansky A, Vouimba RM, Cohen H, Richter-Levin G (2006). Activity and plasticity in the CA1, the dentate gyrus, and the amygdala following controllable vs. uncontrollable water stress. *Hippocampus*.

[27] Vouimba RM, Yaniv D, Diamond D, Richter-Levin G (2004). Effects of inescapable stress on LTP in the amygdala versus the dentate gyrus of freely behaving rats. *European Journal of Neuroscience*.

[28] Yaniv D, Vouimba RM, Diamond DM, Richter-Levin G (2003). Simultaneous induction of long-term potentiation in the hippocampus and the amygdala by entorhinal cortex activation: mechanistic and temporal profiles. *Neuroscience*.

[29] Canning KJ, Leung LS (1997). Lateral entorhinal, perirhinal, and amygdala-entorhinal transition projections to hippocampal CA1 and dentate gyrus in the rat: a current source density study. *Hippocampus*.

[30] Cain DP, Boon F, Hargreaves EL (1992). Evidence for different neurochemical contributions to long-term potentiation and to kindling and kindling-induced potentiation: role of NMDA and urethane-sensitive mechanisms. *Experimental Neurology*.

[31] MacIver MB, Tauck DL, Kendig JJ (1989). General anaesthetic modification of synaptic facilitation and long-term potentiation in hippocampus. *British Journal of Anaesthesia*.

[32] Gilbert ME, Mack CM (1999). Field potential recordings in dentate gyrus of anesthetized rats: stability of baseline. *Hippocampus*.

[33] Shors TJ, Dryver E (1994). Effect of stress and long-term potentiation (LTP) on subsequent LTP and the theta burst response in the dentate gyrus. *Brain Research*.

[34] Abraham WC, Mason-Parker SE, Bear MF, Webb S, Tate WP (2001). Heterosynaptic metaplasticity in the hippocampus in vivo: a BCM-like modifiable threshold for LTP. *Proceedings of the National Academy of Sciences of the United States of America*.

[35] Maren S (1995). Sexually dimorphic perforant path long-term potentiation (LTP) in urethane-anesthetized rats. *Neuroscience Letters*.

[36] Freudenthal R, Romano A, Routtenberg A (2004). Transcription factor NF-*κ*B activation after in vivo perforant path LTP in mouse hippocampus. *Hippocampus*.

[37] Blaise JH, Koranda JL, Chow U, Haines KE, Dorward EC (2008). Neonatal isolation stress alters bidirectional long-term synaptic plasticity in amygdalo-hippocampal synapses in freely behaving adult rats. *Brain Research*.

[38] Hargreaves EL, Cain DP, Vanderwolf CH (1990). Learning and behavioral-long-term potentiation: importance of controlling for motor activity. *Journal of Neuroscience*.

[39] Winson J, Abzug C (1978). Neuronal transmission through hippocampal pathways dependent on behavior. *Journal of Neurophysiology*.

[40] Aroniadou-Anderjaska V, Post RM, Rogawski MA, Li H (2001). Input-specific LTP and depotentiation in the basolateral amygdala. *NeuroReport*.

[41] Stanford SC (1995). Central noradrenergic neurones and stress. *Pharmacology and Therapeutics*.

[42] Galvez R, Mesches MH, Mcgaugh JL (1996). Norepinephrine release in the amygdala in response to footshock stimulation. *Neurobiology of Learning and Memory*.

[43] Quirarte GL, Galvez R, Roozendaal B, McGaugh JL (1998). Norepinephrine release in the amygdala in response to footshock and opioid peptidergic drugs. *Brain Research*.

[44] Tanaka M, Yoshida M, Emoto H, Ishii H (2000). Noradrenaline systems in the hypothalamus, amygdala and locus coeruleus are involved in the provocation of anxiety: basic studies. *European Journal of Pharmacology*.

[45] Aroniadou-Anderjaska V, Qashu F, Braga MFM (2007). Mechanisms regulating GABAergic inhibitory transmission in the basolateral amygdala: implications for epilepsy and anxiety disorders. *Amino Acids*.

[46] Berlau DJ, McGaugh JL (2006). Enhancement of extinction memory consolidation: the role of the noradrenergic and GABAergic systems within the basolateral amygdala. *Neurobiology of Learning and Memory*.

[47] McDonald AJ, Mascagni F (1997). Projections of the lateral entorhinal cortex to the amygdala: a Phaseolus vulgaris leucoagglutinin study in the rat. *Neuroscience*.

[48] Maren S, Fanselow MS (1995). Synaptic plasticity in the basolateral amygdala induced by hippocampal formation stimulation in vivo. *Journal of Neuroscience*.

